# *Bacillus Calmette-Guérin* Induces PD-L1 Expression on Antigen-Presenting Cells via Autocrine and Paracrine Interleukin-STAT3 Circuits

**DOI:** 10.1038/s41598-019-40145-0

**Published:** 2019-03-06

**Authors:** Alastair Copland, Adam Sparrow, Peter Hart, Gil Reynolds Diogo, Mathew Paul, Miyuki Azuma, Rajko Reljic

**Affiliations:** 1 Infection and Immunity, St George’s Medical School, University of London, London, UK; 2 Department of Molecular Immunology, Tokyo Medical and Dental University (TMDU), Tokyo, Japan; 3 Institute of Immunology and Immunotherapy, College of Medical and Dental Sciences, University of Birmingham, Birmingham, UK

## Abstract

*Bacillus Calmette-Guérin* (BCG) is the only licensed vaccine for tuberculosis (TB), and is also used as an immunotherapy for bladder cancer and other malignancies due to its immunostimulatory properties. Mycobacteria spp., however, are well known for their numerous immune evasion mechanisms that limit the true potential of their therapeutic use. One such major mechanism is the induction of programmed death ligand-1 (PD-L1), which mitigates adaptive immune responses. Here, we sought to unravel the molecular pathways behind PD-L1 up-regulation on antigen-presenting cells (APCs) by BCG. We found that infection of APCs with BCG induced PD-L1 up-regulation, but that this did not depend on direct infection, suggesting a soluble mediator for this effect. BCG induced potent quantities of IL-6 and IL-10, and the downstream transcription factor STAT3 was hyper-phosphorylated. Intracellular analyses revealed that levels of PD-L1 molecules were associated with the STAT3 phosphorylation state, suggesting a causal link. Neutralisation of the IL-6 or IL-10 cytokine receptors dampened STAT3 phosphorylation and BCG-mediated up-regulation of PD-L1 on APCs. Pharmacological inhibition of STAT3 achieved the same effect, confirming an autocrine-paracrine cytokine loop as a mechanism for BCG-mediated up-regulation of PD-L1. Finally, an *in vivo* immunisation model showed that BCG vaccination under PD-L1 blockade could enhance antigen-specific memory CD4 T-cell responses. These novel findings could lead to refinement of BCG as both a vaccine for infectious disease and as a cancer immunotherapy.

## Introduction

The correct balance of immune effector and regulatory responses depends on a number of molecular interactions between the antigen-presenting cell (APC) and T-cell. A key interaction for immunological tolerance is between the receptors programmed death-ligand 1 (PD-L1) and programmed death-1 (PD-1). APC expression of PD-L1 leads to binding of this molecule to PD-1 on T-cells, resulting in activation of the tyrosine phosphatase SHP-2 and dephosphorylation of critical kinases involved in T-cell receptor (TCR) signalling. Blockade of this interaction diminishes Treg frequencies^1^, enhances Th1 and Th17 effector cell frequencies^2^ and increases cytokine production both *in vitro* and *in vivo*^3^. The PD-L1:PD-1 interaction has thus been targeted in immunological situations that feature restricted antigen presentation, T-cell anergy and immune tolerance as detrimental characteristics – namely chronic infectious diseases and malignancies. In the latter scenario, clinical trials have demonstrated the remarkable efficacy of drugs developed to target these receptors, with up to 87% clinical response rates in some refractory cancers^4^.

Worldwide, tuberculosis (TB) is the leading cause of death due to infectious disease. The only vaccine available is *Bacillus Calmette-Guérin* (BCG), which shows only modest protection in adults and alarmingly low efficacy in developing countries, where TB mortality is highest. BCG (like its pathogenic relative, *M*. *tuberculosis*) can impede antigen presentation *in vivo*^5,6^, which is believed to contribute to the relatively low efficacy of the vaccine in humans. While mycobacteria-induced PD-L1 expression has been postulated as a major mechanism driving impaired antigen presentation^7,8^, it is currently not fully understood (i) the molecular mechanisms underpinning BCG-mediated PD-L1 up-regulation, and (ii) the immunological consequences of blocking this pathway during BCG immunisation.

For over 30 years, BCG has been employed as a front-line immunotherapy for bladder cancer^9^, and has been used since the late 1960s for malignant melanoma^10^. Although the mechanism of action remains to be completely elucidated, it is believed that the bacteria trigger an inflammatory response that leads to immune cell infiltration of the tumour site, thus facilitating immune-mediated clearance^11^. This is likely to be mediated by innate (i.e. Toll-like receptor) signalling, providing scope for improvement by concomitant engagement of the adaptive immune responses, which are known to be suppressed by PD-L1.

Here, we show for the first time that BCG can induce the up-regulation of PD-L1 on both macrophages and dendritic cells (DCs) via autocrine/paracrine secretion of STAT3-activating cytokines, chiefly IL-6 and IL-10. Blockade of the PD-L1 receptor *in vivo* during BCG immunisation led to superior CD4 T-cell responses to recall antigen, thus highlighting the potential utility of this pathway in clinical settings. These findings provide new targets for improving BCG as both a TB vaccine and cancer immunotherapy.

## Materials and Methods

### Ethics

All experiments involving live animals had full ethical approval from St George’s University ethical committee, under UK Home Office project license 70/7490, according to the Animals in Scientific Procedures Act, 1986.

### Bacteria

BCG strain Pasteur was a kind gift from Professor Juraj Ivanyi (King’s College, London) and was grown according to previous reports^12^, using standard microbiological techniques. BCG expressing green fluorescent protein (GFP; also from the Ivanyi laboratory) was grown in identical conditions, but under selective media and agar containing 50 μg/mL hygromycin B (Sigma-Aldrich).

### Mice and Immunisations

Female C57BL/6 mice (6 to 12 weeks old) were obtained from Charles River laboratories, UK. Mice were administered 1 mg of PD-L1-blocking antibody MIH5^13^ or the rat IgG2a isotype control MAC219^14^ (kind gifts from Professor Anne Cooke, University of Cambridge) via the intraperitoneal (i.p.) route (day -1). Twenty-four hours later (day 0), mice received 1 × 10^6^ CFU BCG subcutaneously (s.c.). Mice then received booster immunisations of MIH5 or MAC219 (1 mg per dose) on days 3, 7 and 14.

**Figure 1:**
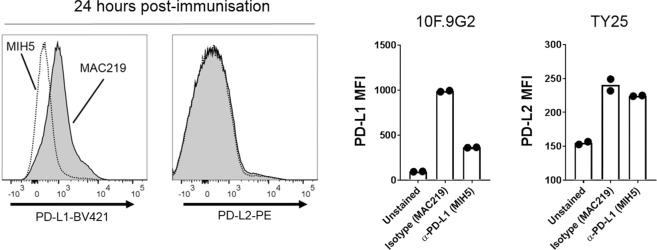
MIH5 blocks the PD-L1 receptor *in vivo*. Mice were immunised with 1 mg mAb via i.p. for 24 h and splenocytes were tested for blockade by staining with a fluorochrome-conjugated competitor clone. *Left*: Representative staining for PD-L1 and PD-L2. *Right:* Combined data from *n* = 2 mice per group. Bars depict means, dots depict individual mice.

To confirm receptor blockade, mice were administered 1 mg MIH5 or MAC219 via the i.p. route, followed by euthanasia at 24 h, and immediate *ex vivo* staining of the splenocytes. Cells were stained with a reported competing fluorochrome-conjugated α-PD-L1 clone (10F.9G2)^15^, which binds to the same epitope as MIH5, to test for successful receptor blockade (Fig. 1). As an additional control for specificity, PD-L2 was also stained after MIH5 or MAC219 treatments.

### Antigen-Presenting Cell Stimulation and Infection

Bone marrow-derived DCs were obtained as previously described^12^. Cells were >90% CD11c^+^ by flow cytometry. DCs were stimulated in complete RPMI (RPMI-1640 containing 10% FCS, 2mM L-glutamine, and 50 μM β-mercaptoethanol ± 10 U/mL penicillin/streptomycin – all from Sigma-Aldrich). For experiments involving macrophages, the cell line J774.1 was used, and cells were grown and stimulated in complete DMEM (same recipe as RPMI – Sigma-Aldrich).

For BCG infections, bacteria were washed in complete media without antibiotics, and then APCs were inoculated at the designated MOI. Cells were cultured in a 5% CO_2_ humidified incubator at 37 °C. In some experiments, *E*. *coli*-derived lipopolysaccharide (LPS; Sigma-Aldrich) was used at 100 ng/mL. Cytokines (Peprotech, UK) were diluted in complete RPMI before administration. Interleukin blocking antibodies (purchased from Biolegend, UK) were pre-cultured with the cells for 2 h before stimulation. Stattic (Tocris Bioscience, UK) was diluted in DMSO (Sigma-Aldrich) and cells were treated for 2 h before infection.

### *Ex vivo* immunogenicity assays

Spleens were aseptically removed from euthanised mice, mechanically homogenised and treated with ACK lysis buffer. Cells were then counted and seeded at 1.5 × 10^6^ per well in complete RPMI, followed by treatment with 10 μg/mL brefeldin A (Sigma-Aldrich). Cells were stimulated with 5 μg/mL Ag85B/Acr (Lionex, Germany) or PPD (NIBSC, UK) with 2 μg/mL α-CD28 (Biolegend) for 6 hours before staining for flow cytometry. PMA/ionomycin treatment (200 ng/mL and 1 μg/mL, respectively – Sigma-Aldrich) was used as a positive control and for staining boundaries (data not shown).

For lymph node analysis, inguinal lymph nodes were excised from euthanised mice on the indicated day, followed by mechanical disruption, counting and immediate flow cytometric analysis.

### Flow cytometry

In most experiments, cells were first washed in PBS and then incubated with 1:1000 viability dye (eFluor780 Fixable Viability Dye; eBioscience) under Fc receptor blockade (1:500 TruStain; Biolegend) for 15–20 minutes. Cells were then washed in flow cytometry buffer (PBS (Invitrogen) containing 0.5% BSA and 0.1% sodium azide – both from Sigma-Aldrich) and stained with the appropriate pre-titrated flow cytometry antibodies for 30 m at 4 °C. Cells were sometimes fixed using Biolegend Fixative Buffer before being acquired on a BD FACSCanto II instrument and analysed using FlowJo software. For assessing phosphorylated residues, cells were instead mildly fixed with Fixative Buffer for 10 m at 37 °C, and then permeabilised with a commercial methanol buffer (True-Phos buffer - Biolegend) for 1 hour before staining, as described elsewhere^16^. For intracellular cytokine staining, cells were fixed with Fixative Buffer, followed by permeabilisation with flow cytometry buffer containing 0.5% saponin (Sigma-Aldrich). Compensation was performed using eBioscience UltraComp beads according to the manufacturer’s instructions. Antibodies used were PD-L1-Brilliant Violet 421, PD-L2-PE, CD11c-PerCP/Cy5.5, CD3ε-FITC, CD4-PerCP/Cy5.5, CD8α-Brilliant Violet 510, IL-2-PE, IL-17A-PE/Cy7, IFN-γ-Brilliant Violet 421, MHC Class II- Brilliant Violet 510, TNF-α-APC and p-STAT3-Alexa Fluor 647. All antibodies were purchased from Biolegend, UK unless otherwise specified.

### Enzyme-linked immunosorbent assay

Cytokine levels in supernatants were measured using eBioscience Ready-Set-Go ELISA kits, according to the manufacturer’s instructions. Plates were read at 482 nm on a Tecan200 plate reader.

### Statistical analysis

For all experiments, statistical tests were performed with Microsoft Excel and GraphPad Prism software, using averaged technical replicates where possible. Each statistical test and post-test is detailed in the relevant figure legends. A *p* value less than 0.05 was considered significant.

## Results

### BCG Induces Up-regulation of PD-L1 Expression on both Macrophages and Dendritic Cells

**Figure 2:**
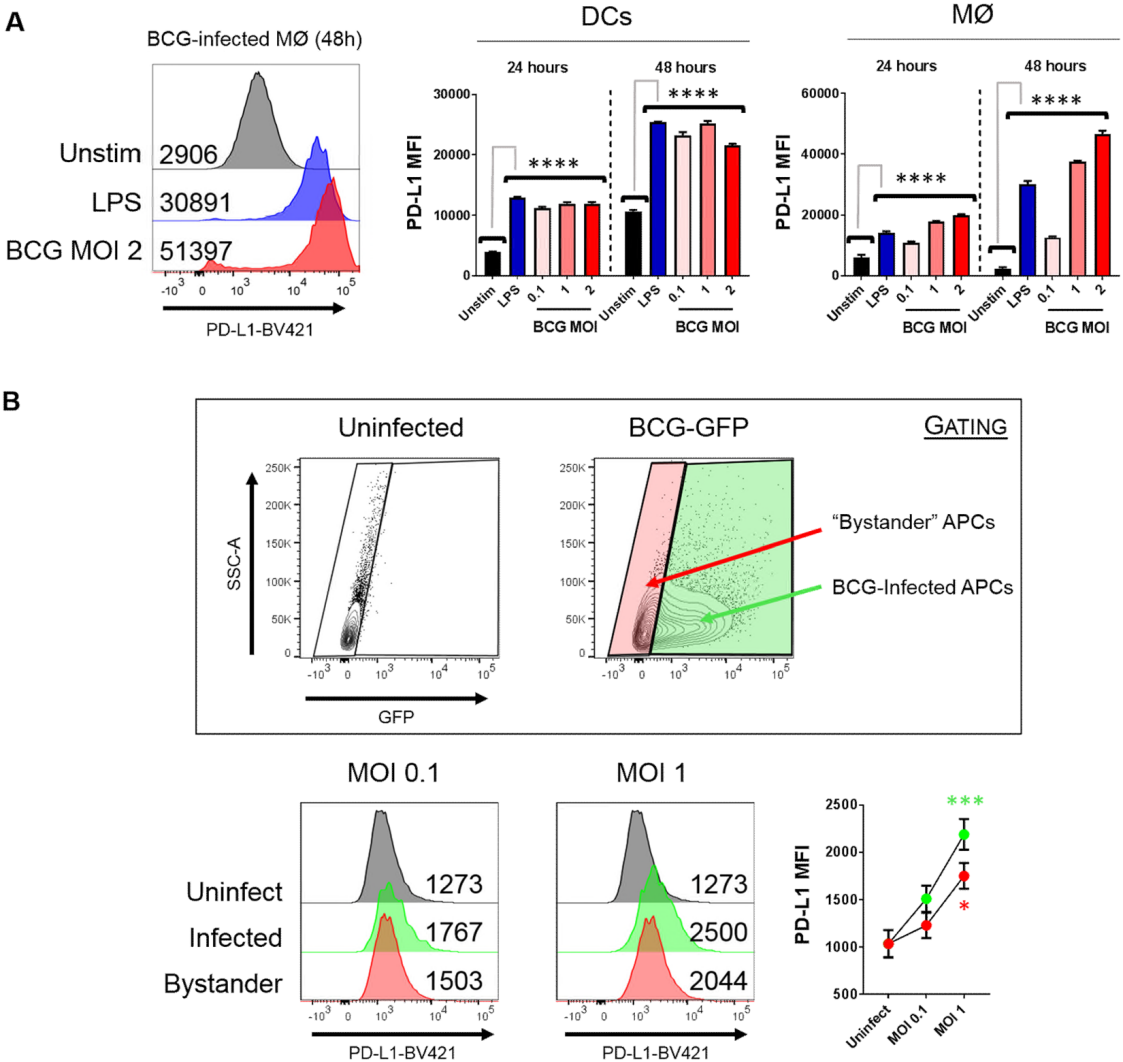
BCG can up-regulate PD-L1 expression without direct cellular association. DCs and macrophages were infected in duplicate with BCG and PD-L1 expression was assessed by flow cytometry. **(A)** *Left:* Representative staining of PD-L1 on macrophages at 48 hours. Cells were gated by size and then viability. Numbers denote median fluorescence intensity (MFI). *Right:* PD-L1 expression on DCs and macrophages infected with 0.1–2 MOI BCG or stimulated with 100 ng/mL LPS, at 24 h and 48 h post-infection. **(B)** Macrophages were infected with BCG expressing GFP for 24 h. *Top*: Gates were set on the negative control to determine GFP^neg^ and GFP^pos^ populations. *Bottom left*: Representative histograms depicting PD-L1 expression in GFP^neg^ and GFP^pos^ cells. *Bottom right:* Pooled data showing PD-L1 MFI in infected and bystander macrophages. Significance was tested against the negative control by two-way ANOVA with Dunnett’s post-test (**A,B**). Bars depict means ± SEM. Data derived from *n* = 3 experiments: ****p < 0.0001. *p < 0.05.

DCs and macrophages are critical for the initiation of adaptive immunity. BCG can induce the up-regulation of PD-L1 expression on pulmonary DCs in mice^7^, however it is unclear whether the same holds true for macrophages. DCs and macrophages were therefore infected with BCG over a range of multiplicities of infection (MOI) and across two time-points (24 h and 48 h); surface expression of PD-L1 was assessed by flow cytometry. As shown in Fig. 2A, the positive control lipopolysaccharide (LPS) was able to significantly increase PD-L1 expression on both cell types (*p* < 0.0001), with a striking >10 fold increase in macrophages at 48 h. Upon infection with BCG, both APC types expressed high levels of PD-L1 compared to the unstimulated control at 24 h and 48 h (*p* < 0.0001), and a dose trend was observed for increasing MOI in macrophages at 48 h.

Next, a transgenic strain of BCG that expresses green fluorescent protein (GFP) was used to determine whether the up-regulation of PD-L1 depended on direct interaction between APCs and the bacteria. Cells were gated by GFP fluorescence into GFP^neg^ (i.e. “bystander”) and GFP^pos^ (i.e. infected) populations. These were then tested for PD-L1 expression. As anticipated, GFP^pos^ infected cells displayed increased expression of PD-L1 that was proportionate to the infectious dose (Fig. 2B). Surprisingly, however, GFP^neg^ bystander cells also exhibited similar dose-dependent increases in PD-L1 expression to GFP^pos^ cells, which was significantly up-regulated compared to the uninfected control at the highest MOI (*p* < 0.05), and approximately 80% that of directly infected cells. In support of these observations, 0.2 µm filtration of BCG infection supernatants, when applied to new cells, was able to up-regulate PD-L1 (Supplementary Fig. 1). As expected, control supernatants from uninfected cells did not affect PD-L1 expression. These data strongly suggested that a soluble, secreted factor in culture was driving PD-L1 up-regulation by BCG.

Interestingly, BCG was able to up-regulate other members of the B7 family (CD80, CD86, PD-L2) to a certain degree, but with a pattern of skewed up-regulation of PD-L1 compared to CD86 (Supplementary Fig. 2).

### BCG Induces IL-6 and IL-10 Production in tandem with STAT3 Phosphorylation

The murine PD-L1 gene (*Cd274*) is under the control of complex regulatory networks and can be induced by a number of inflammatory cytokines or TLR ligands^17^. Many of these control mechanisms are cell type-specific. Since mycobacteria are adept at driving STAT3 activation^18^, and since STAT3 is capable of binding to the PD-L1 promoter in tolerogenic DCs^19^, we hypothesised that this signalling pathway was mediating the observed effects of infection.

**Figure 3:**
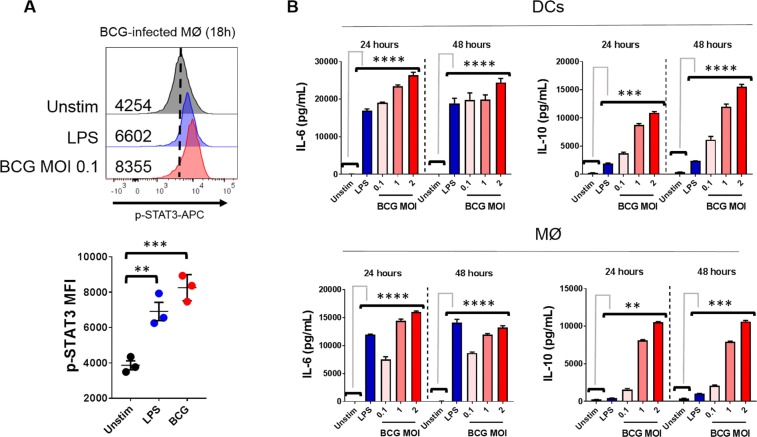
BCG induces STAT3 activation and secretion of STAT3-activating interleukins. (**A)** Macrophages were infected in duplicate for 18 h with 0.1 MOI BCG or 100 ng/mL LPS. Cells were stained for surface PD-L1 expression, followed by intracellular staining for p-STAT3. *Top*: Representative histogram showing levels of STAT3 phosphorylation. Numbers denote MFI. *Bottom*: Pooled data from several experiments showing p-STAT3 MFI. **(B)** DCs and macrophages were infected in duplicate with 0.1–2 MOI BCG or 100 ng/mL LPS for 24–28 h and supernatants were tested for IL-6 and IL-10 by ELISA. Significance was tested by one-way ANOVA with Tukey’s post-test (**A**) or two-way ANOVA with Dunnett’s post-test against the negative control (**B**). Data are derived from *n* = 3 experiments. Bars depict means ± SEM. ****p < 0.0001, ***p < 0.001, **p < 0.01.

DCs and macrophages were infected for 18 h and intracellular flow cytometry was used to determine the levels of STAT3 tyrosine residue 705 phosphorylation. As can be observed in Fig. 3A, BCG was able to hyper-phosphorylate the STAT3 transcription factor compared to unstimulated cells (*p* < 0.001). Notably, there was a trend for increased phosphorylation when comparing BCG to the positive control LPS. We next hypothesised that APCs were producing STAT3-activating cytokines. STAT3 can be activated weakly by IL-2 and IL-12, but strongly by prototypical myeloid-type cytokines such as IL-6 and IL-10. APCs were therefore infected with BCG (or stimulated with LPS) for 24–48 h and levels of IL-6 and IL-10 were measured by ELISA. BCG was found to induce potent quantities of IL-6 in both DCs and macrophages at a range of MOI (Fig. 3B; *p* < 0.0001 BCG vs unstimulated cells). BCG elicited IL-10 in both cell types. Strikingly, with regards to IL-10 production, BCG greatly surpassed the ability of LPS at MOI = 2, with over 4-fold concentrations of this cytokine compared to the positive control.

### PD-L1 Expression Correlates with STAT3 Phosphorylation

**Figure 4:**
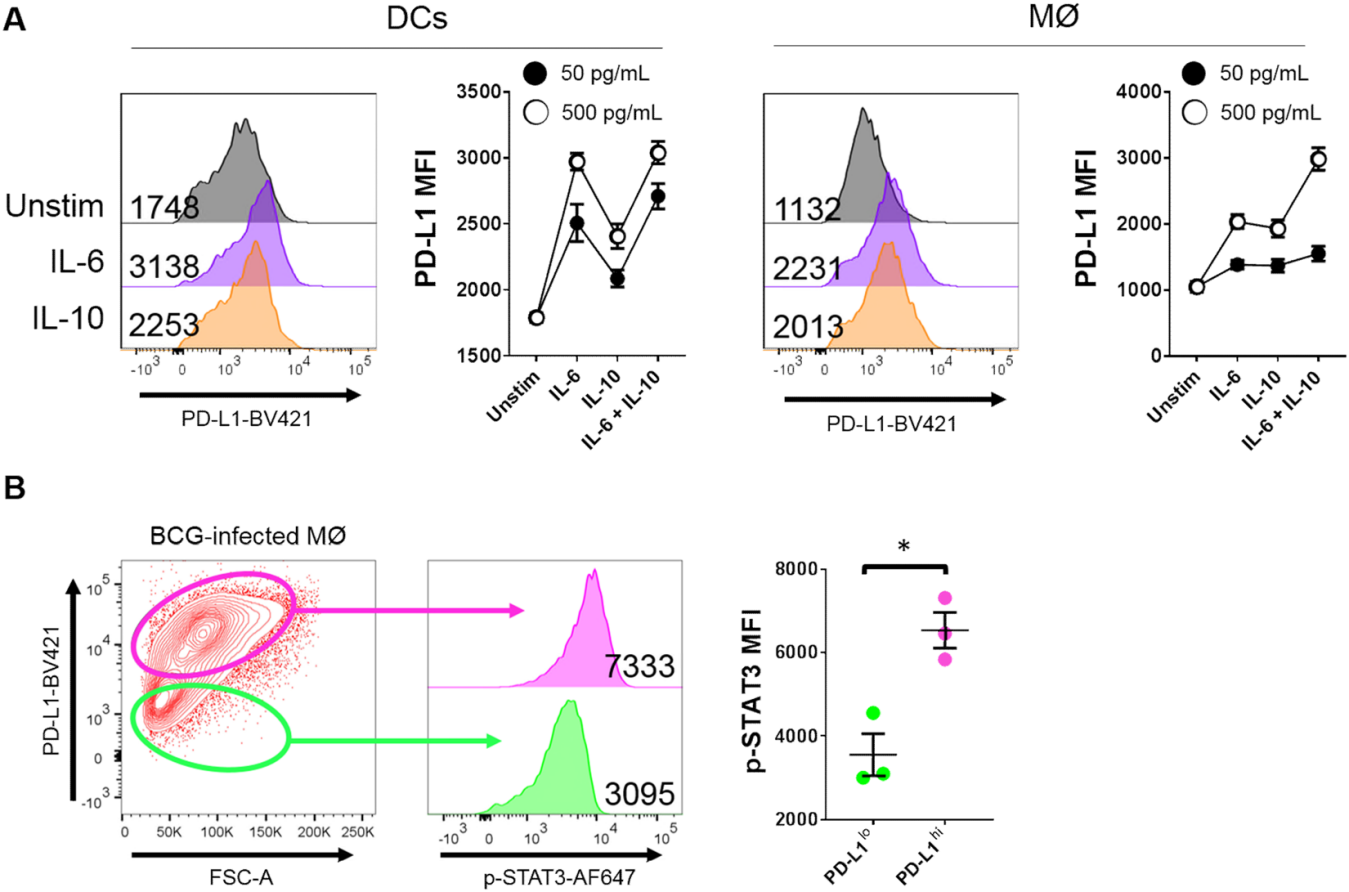
IL-6 and IL-10 are sufficient for PD-L1 expression and STAT3 is associated with its expression. (**A)** DCs and macrophages were stimulated with 50 or 500 pg/mL recombinant IL-6, IL-10 or both for 24 h and PD-L1 expression was assessed by flow cytometry. Representative histograms are depicted with MFIs for the relevant conditions, alongside pooled data from multiple experiments. **(B)** Macrophages were infected with 0.1 MOI BCG for 18 h and p-STAT3 was analysed by flow cytometry. *Left*: Representative histogram with MFIs and gating strategy, showing p-STAT3 analysis within PD-L1^lo^ and PD-L1^hi^ populations. *Right*: Pooled data from multiple experiments. Data are derived from *n* = 3 experiments (**A, B**). Significance was tested by student’s t-test (**B**). Bars depict means ± SEM. *p < 0.05.

With the hypothesis that BCG was inducing STAT3-activating cytokines to up-regulate PD-L1, we treated DCs and macrophages with different concentrations of IL-6 and IL-10 and measured PD-L1 up-regulation by flow cytometry. In DCs (Fig. 4A), IL-6 played a dominant role in the up-regulation of PD-L1 expression compared to IL-10. When both cytokines were used in combination, there was only a marginal increase above the levels of PD-L1 expression conferred by IL-6. In macrophages, by contrast, IL-6 and IL-10 behaved similarly in terms of receptor up-regulation, and there was a combinatorial effect that was clearly evident at 500 pg/mL. For both cell types, there was a dose-dependent effect, with 500 pg/mL of any cytokine (or combination) being superior to 50 pg/mL in up-regulating PD-L1 expression.

Next, macrophages were infected with BCG for 18 h and cells were then permeabilised as before and co-stained for p-STAT3 and PD-L1. Cells were then divided into PD-L1^lo^ and PD-L1^hi^ populations and then levels of p-STAT3 were quantified. As shown in Fig. 4B, PD-L1^lo^ cells exhibited significantly lower levels of STAT3 phosphorylation compared to their PD-L1^hi^ counterparts (*p* < 0.05), with a doubling of fluorescence intensity in some experiments. Taken together, these data suggested that BCG was utilising interleukin signalling pathways in order to up-regulate expression of PD-L1.

### IL-6R/IL-10R Blockade or STAT3 Inhibition Leads to Impeded PD-L1 Up-regulation by BCG

We next questioned whether direct intervention in the interleukin-STAT3 axis could revert the up-regulation of PD-L1 by BCG. To this end, we employed a combination of monoclonal antibodies (mAbs) that are known to block the IL-6 and IL-10 cytokine receptors, alongside the pharmacological inhibitor ‘Stattic’: a well-characterised and highly specific small-molecule inhibitor of STAT3 transcriptional activity^20^.

**Figure 5:**
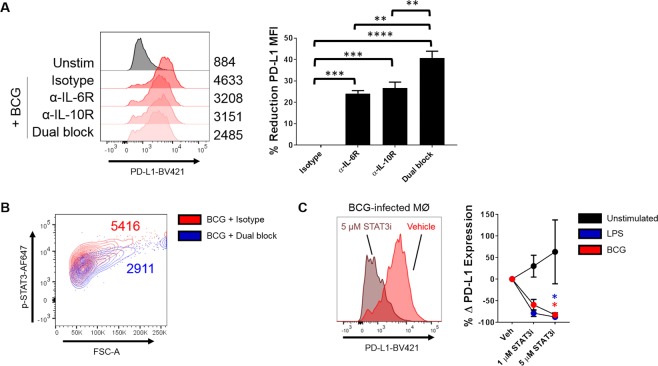
BCG-mediated up-regulation of PD-L1 is diminished by inhibiting the interleukin-STAT3 axis. (**A)** Macrophages were pre-treated with 50 μg/mL blocking antibodies or isotype controls for ~2 h and infected with 0.1 MOI BCG for 24 h. PD-L1 expression was measured by flow cytometry. *Left*: Representative histogram of PD-L1 expression with MFIs depicted. *Right*: Pooled data from multiple experiments, with calculated percentage reduction in PD-L1 MFI. **(B)** Macrophages were pre-treated with 50 μg/mL IL-6R and IL-10R blocking antibodies and then infected with 0.1 MOI BCG for 18 h. Levels of p-STAT3 were determined by intracellular flow cytometry. Shown is a representative plot with depicted MFI for p-STAT3. **(C)** Macrophages were pre-treated with the indicated dose of Stattic or DMSO equivalent for 2 hours, and then infected with 0.1 MOI BCG or stimulated with 100 ng/mL LPS for 24 h. Levels of PD-L1 were measured by flow cytometry. *Left*: Representative histogram showing Stattic-mediated PD-L1 down-regulation. *Right:* Pooled data from multiple experiments showing percentage change from the vehicle control. Significance was tested by one-way ANOVA with Tukey’s post-test (**A**) or two-way ANOVA with Dunnett’s post-test vs the vehicle control (**B**). Bars depict means ± SEM. Data are derived from *n* = 3 experiments. ****p < 0.0001, ***p < 0.001, **p < 0.01, *p < 0.05.

To test whether neutralising the biological activities of endogenous cytokines could reduce the increase in PD-L1 expression caused by BCG, cells were first pre-incubated under IL-6R (mAb D7715A7) or IL-10R (mAb 1B1.3a) blockade, or a combination of both, for 2 h. Isotype controls served as controls for non-specific activity. Cells were then infected with BCG and after 24 h, PD-L1 expression was determined (Fig. 5A). Both IL-6 and IL-10 blockade significantly reduced PD-L1 expression (*p* < 0.001) compared to the isotype control baseline. As expected, the combination of blocking both receptors led to the greatest reduction in PD-L1 fluorescence (~40–45%; *p* < 0.0001 vs isotype control). In keeping with the hypothesised role of STAT3 in BCG-induced PD-L1 expression, blockade of both receptors also led to a large reduction in STAT3 phosphorylation, as shown in Fig. 5B (BCG + isotype MFI: 5416; BCG + dual blockade MFI: 2911).

To confirm that STAT3 was mediating the up-regulation of PD-L1 expression by BCG, cells were then pre-treated with Stattic or a vehicle control for 2 hours before infection with a low dose of bacteria (Fig. 5C). LPS served as a positive control, since it can induce PD-L1 expression via this pathway^21^. After 24 hours, the cells treated with 5 μM Stattic and infected with BCG showed a dramatically reduced expression level of PD-L1 compared to those treated with a vehicle control (*p* < 0.05), with an near-100% reduction in PD-L1 expression. Similar results were observed for LPS. Strikingly, resting cells treated with Stattic actually increased PD-L1 expression compared to the vehicle control, although this was a non-significant trend with high variation. Collectively, these data proved that BCG could modulate PD-L1 expression by interleukin-STAT3 signalling circuits.

### *In vivo* PD-L1 Blockade Augments Antigen-Specific Memory CD4 T-cell Responses

**Figure 6:**
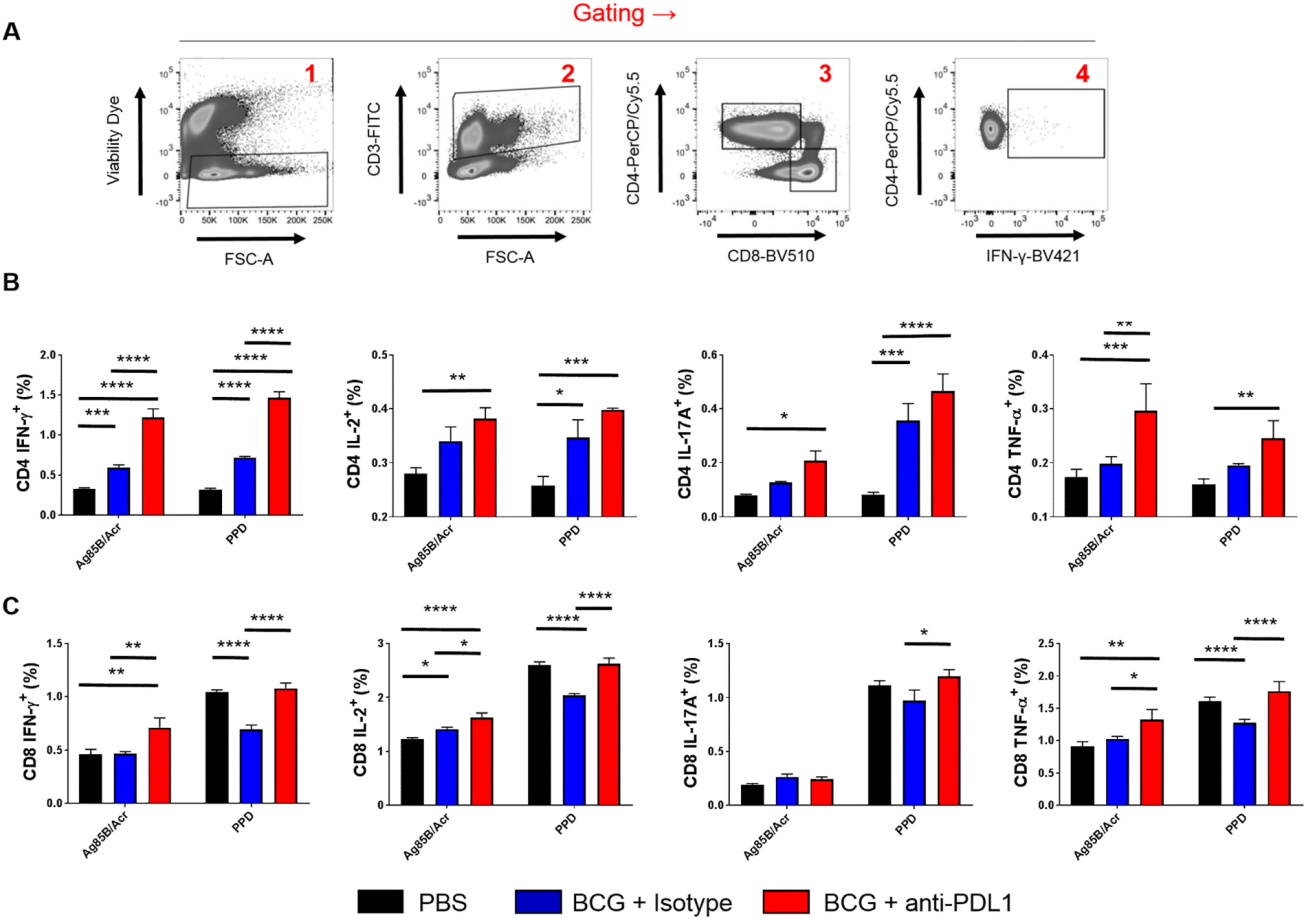
*In vivo* PD-L1 blockade during BCG immunisation boosts CD4 T-cell cytokine production. Splenocytes from immunised mice were cultured in duplicate with 2 μg/mL α-CD28 and either Ag85B/Acr (5 μg/mL) or PPD (5 μg/mL) for 6 hours, in the presence of brefeldin A (10 μg/mL). **(A)** Cells were then stained for Th1-Th17 cytokines and gated by size/viability → CD3^+^  → CD4^+^/CD8^+^. **(B)** CD4 T-cell cytokine responses. **(C)** CD8 T-cell cytokine responses. Significance was tested by two-way ANOVA with Dunnett’s post-test. Data are derived from *n* = 3 mice per group. Bars depict means ± SEM. ****p < 0.0001, ***p < 0.001, **p < 0.01, *p < 0.05.

A proof-of-principle *in vivo* immunogenicity assay was next performed in order to establish that targeting the PD-L1 receptor during BCG immunisation could lead to increased T-cell function. A cytokine panel spanning several hallmark Th1/Th17 cytokines (IFN-γ, IL-2, IL-17A, TNF-α) was used to determine T-cell reactogenicity in a vaccination model with recall mycobacterial antigens (Fig. 6A).

First, the up-regulation of PD-L1 *in vivo* was determined by sub-cutaneous immunisation with BCG followed by characterisation of PD-L1 expression in the draining lymph nodes (Supplementary Fig. 3). It was found that BCG was able to up-regulate PD-L1 in MHC Class II^high^ CD11c^+^ DCs, but unable to do the same in T-cells, confirming a specific effect in APCs. Next, mice were treated with 1 mg of the PD-L1 blocking antibody MIH5 via the intraperitoneal route, followed by immunisation with subcutaneous BCG. Mice were then given follow-up booster immunisations to maintain the PD-L1 blockade. An isotype control was used at the same concentration to control for non-specific effects. On day 21, splenocytes from immunised mice were then pulsed with either immunodominant and latency-associated antigens (Ag85B and Acr, respectively) or a mixture of protein antigens (PPD). As shown in Fig. 6B, there was a general trend for an increase in antigen-specific cytokine production in CD4 T-cells. Under PD-L1 blockade, BCG induced significantly more IFN-γ in response to Ag85B/Acr and PPD (IFN-γ: *p* < 0.0001), compared to the isotype control. TNF-α production was also increased in response to Ag85B/Acr (*p* < 0.01). Regarding IL-2 and IL-17A, there were non-significant increases caused by PD-L1 blockade under both antigen recall conditions.

Turning our attention to the CD8 T-cell compartment (Fig. 6C), we found that BCG was much poorer at inducing antigen-specific cytokine responses, as has been observed previously by others^22^. With the exception of IL-2 after Ag85B/Acr pulsing (*p* < 0.05), splenocytes from mice that received BCG + isotype control were unable to produce more cytokines than splenocytes from mock-immunised mice. Only two cytokines were found to be increased by PD-L1 blockade beyond the PBS control group in response to Ag85B/Acr but not PPD: IFN-γ (*p* < 0.01) and TNF-α (*p* < 0.01), however these effects were marginal. Thus we concluded that PD-L1 blockade can effectively boost CD4-dependent T-cell immunity, with only marginal effects on boosting CD8 T-cell responses.

## Discussion

It is long-recognised that bacteria (and indeed other pathogens) belonging to genetically distinct phyla are capable of modulating the repertoire of co-stimulatory and co-inhibitory molecules expressed on the APC surface^23^, thus affirming the critical importance of said receptors in the directing of adaptive immune responses. Mycobacteria represent an example of immune evasion *par excellence* due to their ancient history of co-evolution with mammalian immune systems^24^; indeed, virtually all pathogenic mycobacteria are obligate parasites, requiring intracellular resources in order to thrive and propagate. It is therefore not surprising that they are biologically equipped to effectively counter adaptive immune responses that would lead to their own clearance.

A centrally important molecule in the control of T-cell immunity is PD-L1. Mice infected with mycobacteria harbour pulmonary DCs that express high levels of PD-L1 and restrict antigen presentation to CD4 T-cells^25^, and PD-L1 blockade in blood and lung lavage from TB patients can enhance responses to Mtb antigens, as seen by greater cytokine production and T-cell proliferation^26,27^. Furthermore, PD-L1 blockade is able to rescue these cells from functional exhaustion, as demonstrated by the reversal of T-cell apoptosis^28^. For bladder cancer, the tumour surface is reportedly decorated with high levels of PD-L1 molecules, and the tumour-infiltrating B- and T-cells express high levels of both PD-L1 and PD-1^29,30^. The utilisation of BCG as a prophylactic (TB) or therapeutic (malignancy) treatment for these diseases, combined with a strategy to mitigate the effects of PD-L1, could provide a strong advantage for the efficacy of BCG.

**Figure 7:**
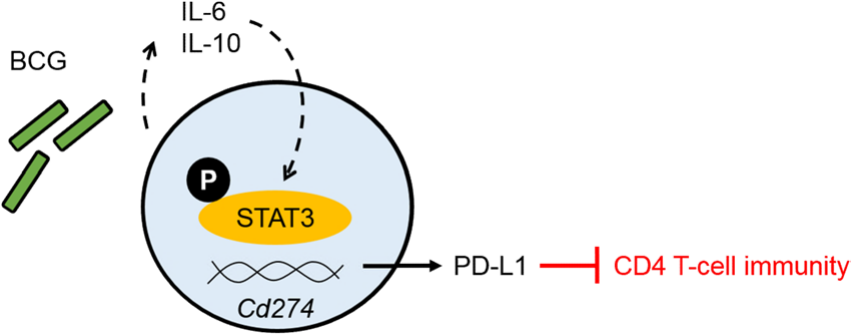
Model for BCG-mediated up-regulation of PD-L1 expression. Proposed schematic showing how BCG targets IL-6 and IL-10 to cause STAT3 phosphorylation and PD-L1 up-regulation.

In this study, for the first time, it was shown that BCG drives up-regulation of PD-L1 expression on APCs by autocrine/paracrine cytokine circuits that lead to STAT3 phosphorylation and up-regulation of this co-inhibitory receptor (illustrated in Fig. 7). It has been known for some time that BCG is a strong inducer of these cytokines via rudimentary TLR signalling, however it was not known that they were driving PD-L1 expression in a biphasic response. The observation that inhibition of STAT3 did not lead to a decrease in PD-L1 expression in resting cells—indeed, there was instead a non-significant increase in expression—is consistent with the notion that the PD-L1 gene promoter requires distinct transcriptional apparatus during the steady-state and during active infection. This is in accord with the fact that DCs and macrophages displayed moderate levels of constitutive PD-L1 expression in the absence of any appreciable cytokine levels^31^. Physiologically, this is undoubtedly to prevent spontaneous activation of the adaptive immune system.

Using the mAb MIH5, we were able to neutralise most of the PD-L1 receptor *in vivo* and augment the quantities of cytokine produced by CD4 T-cells in response to recall antigen in a proof-of-principle experiment. It is interesting to note that PD-L1 blockade *in vivo* did not greatly boost the antigen-specific CD8 T-cell responses beyond that of the mock-immunised control group. BCG is widely known to only minimally induce cytotoxic T-cell responses, with a strong bias towards CD4 responses^22^. This may be due to so-called CD8 ‘decoy antigens’, such as TB10.4, that serve to divert responses away from immunogenic epitopes for cytotoxic T-cells^32^. Whether the enhancement of CD4 T-cell immunogenicity in the absence of strong CD8 T-cell immunogenicity leads to better vaccine (i.e. in the case of TB) or immunotherapeutic (i.e. in the case of a carcinoma) outcomes warrants testing in disease-specific animal models.

An important limitation of this study is that IL-6 and IL-10 are unlikely to constitute the only two cytokines driving the STAT3-PD-L1 pathway, and we expect that there is redundancy in which cytokine drives PD-L1, as long as STAT3 is adequately activated. STAT3 can also be activated by nearly 20 members of the IL-6 and IL-10 cytokine families. However, IL-6 and IL-10 are two of the most abundant cytokines secreted by myeloid cells upon activation, and while dual blockade of the receptors led to a ~45% reduction in PD-L1, this could reflect the fact that blocking antibodies do not completely neutralise all biological activity^33^. STAT3 can also be weakly activated by TLR signalling and Src-family kinases^34,35^, and we cannot exclude the possibility of such contributions to its phosphorylation state. Furthermore, there are undoubtedly other transcriptional co-factors recruited to the *Cd274* promoter alongside STAT3. Regardless, we have conclusively shown that STAT3 is biologically essential for its activity by BCG, and linked this to biological targets that are amenable to intervention in clinical settings (e.g. tocilizumab, an α-IL-6R mAb). Indeed, this study provides four such points of intervention when using BCG: (i) STAT3-activating cytokines, (ii) IL-6/IL-10 cytokine receptors, (iii) the STAT3 transcription factor and (iv) the PD-L1 receptor itself.

STAT3 presents a highly appealing therapeutic target other than its role in PD-L1 expression due to the fact that is a pleiotropic master controller of general tolerance in APCs. STAT3 can suppress autophagy^36^, nitric oxide production^37^, IL-12 production^38^, and co-stimulatory molecule expression^39^. Given our new findings, targeting this pathway during vaccination with BCG (for TB or malignancy) could reap multiple therapeutic benefits.

In conclusion, we have revealed novel molecular insights into how BCG up-regulates PD-L1 on APCs, allowing for improved immunogenicity to specific antigens, but also more intricate understanding of fundamental host-pathogen interactions. Future work will focus on exploring this pathway in specific disease models that rely on BCG as a treatment, with the aim of bolstering immunological parameters, and ultimately, treatment efficacy.

## Supplementary information

Supplementary information
