# Supplementary material for: Bacillus Calmette-Guérin Induces PD-L1 Expression on Antigen-Presenting Cells via Autocrine and Paracrine Interleukin-STAT3 Circuits
Source: Sci Rep. 2019 Mar 6;9:3655. doi: 10.1038/s41598-019-40145-0 (PMC6403281; doi:10.1038/s41598-019-40145-0)
Supplement: Supplementary file 1 — Supplementary information [file 41598_2019_40145_MOESM1_ESM.docx]

**Supplementary Information for the manuscript:**

***Bacillus Calmette-Guérin* Induces PD-L1 Expression on Antigen-Presenting Cells via Autocrine and Paracrine Interleukin-STAT3 Circuits**

Alastair Copland^1,3^, Adam Sparrow^1^, Peter Hart^1^, Gil Reynolds Diogo^1^, Mathew Paul^1^, Miyuki Azuma^2^, Rajko Reljic^1#^

1 Infection and Immunity, St George’s Medical School, University of London, UK

2 Department of Molecular Immunology, Tokyo Medical and Dental University (TMDU), Japan

3 Institute of Immunology and Immunotherapy, College of Medical and Dental Sciences, University of Birmingham, UK

# Corresponding author, [rreljic@sgul.ac.uk](mailto:rreljic@sgul.ac.uk)

This supplementary PDF file includes the following information:

Supplementary Figures S1-3

**
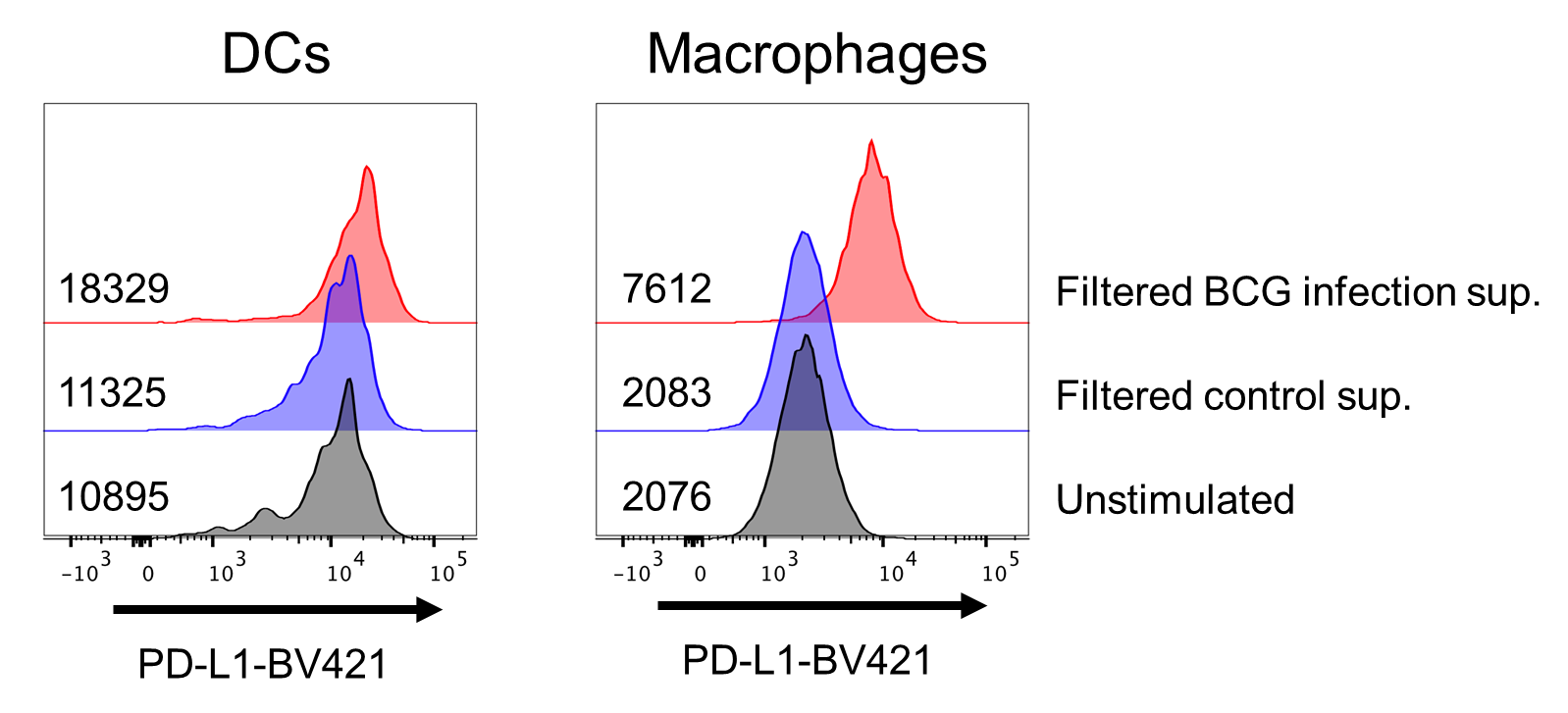
**

**Supplementary Figure 1**. Supernatants from BCG-infected Cultures can Up-regulate PD-L1. DCs or macrophages were infected with BCG at a MOI of 1, followed by 0.2 µm filtration. Supernatants from uninfected cells were used as a control. In fresh cells, supernatants were added in a 1:1 ratio, and after 24 hours, PD-L1 expression was measured by flow cytometry. Data are representative of *n* = 2 replicates.


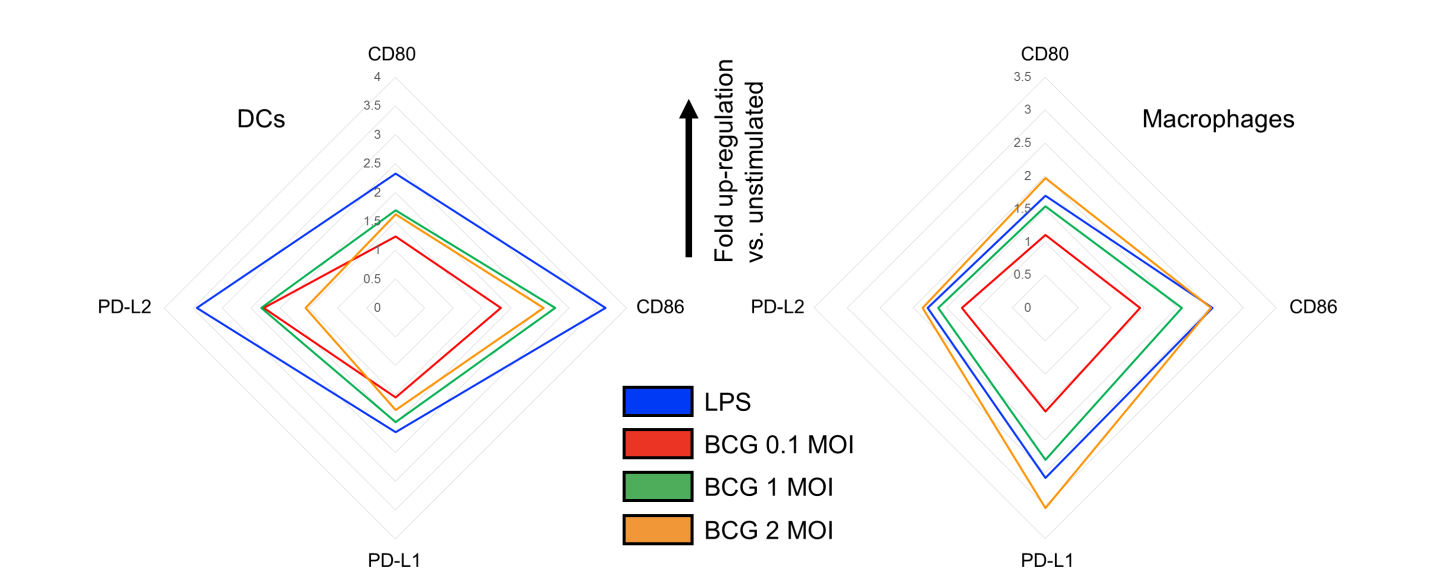


**Supplementary Figure 2**. BCG Up-regulates Multiple B7 Family Members with a Bias Towards PD-L1. DCs or macrophages were infected with 0.1-2 MOI BCG or 100 ng/mL LPS for 24 hours and then assessed for CD80, CD86, PD-L1 or PD-L2 expression. Fold-increase in expression was calculated relative to the unstimulated control, using the formula: $\frac{Stimulated MFI}{Unstimulated MFI} = Fold Upregulation.$ Data are pooled from *n* = 2 replicates.


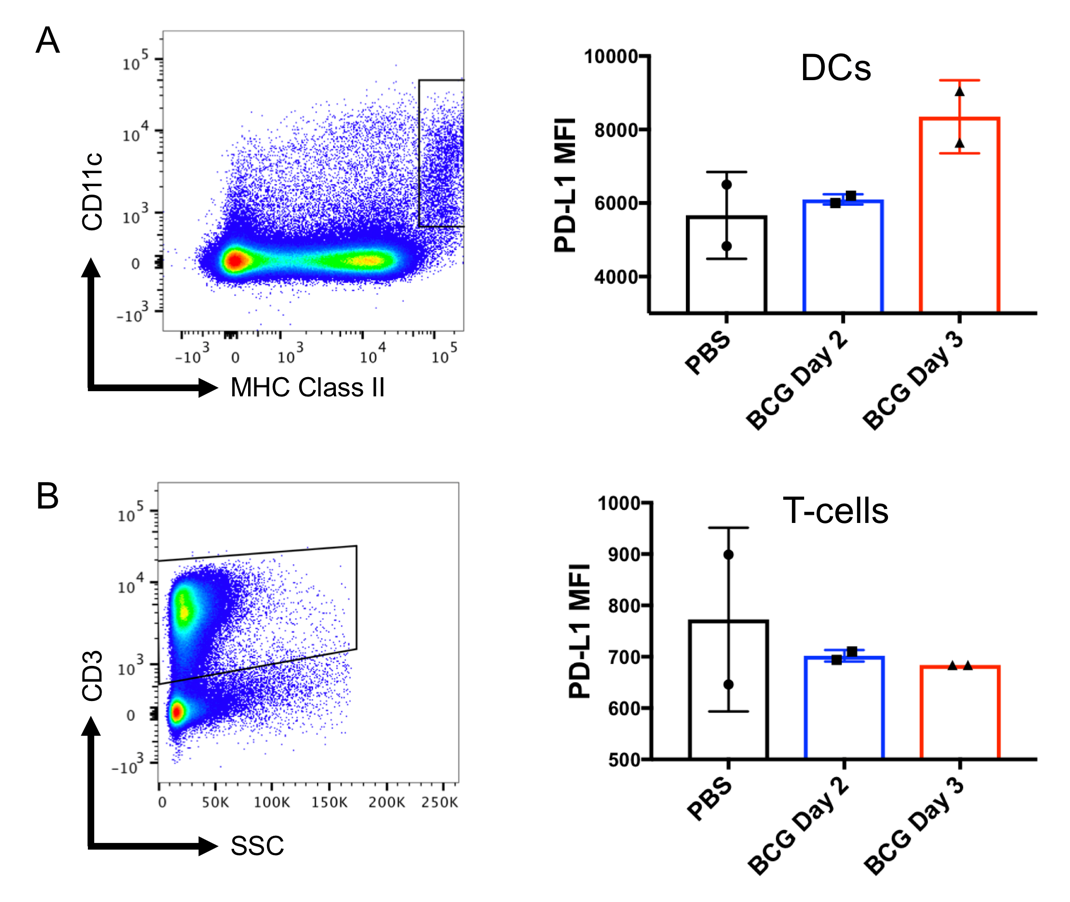


**Supplementary Figure 3.** BCG Induces PD-L1 Up-regulation on DCs In Vivo. Mice were immunised with 1 x 10^6^ CFU BCG subcutaneously, or mock-immunised with PBS, followed by flow cytometric analysis of live single cells from the inguinal lymph node at the indicated time-point. **(A)** *Left:* Gating strategy for analysing DCs (MHC Class II^high^ CD11c^+^). *Right:* PD-L1 expression with indicated MFI values. **(B)** *Left:* Gating strategy for analysing T-cells (CD3^+^). *Right:* PD-L1 expression with indicated MFI values. Data are from *n* = 2 mice.
